# Beyond hepatitis C: clinical spectrum and demographic characteristics of anti-rods and rings antibodies in a large ANA-positive cohort

**DOI:** 10.3389/fimmu.2026.1831506

**Published:** 2026-06-03

**Authors:** Pingping Wang, Shiji Wu, Jingting Liu, Jianying Pei, Chong Zhang, Xing Chen, Xiang Zhang, Yun Wang

**Affiliations:** 1 Clinical Laboratory Center, Gansu Provincial Maternity and Child-Care Hospital, Lanzhou, China; 2 Department of Laboratory Medicine, Tongji Hospital, Tongji Medical College, Huazhong University of Science and Technology, Wuhan, China

**Keywords:** antinuclear antibodies (ANAs), anti-rods and rings (anti-RR), autoimmune diseases, infectious diseases, metabolism

## Abstract

**Background:**

The anti-rods and rings (anti-RR) pattern (AC-23) is traditionally associated with Hepatitis C Virus (HCV) infection and interferon therapy. However, emerging evidence suggests a broader clinical spectrum. This study aims to characterize the clinical associations, demographic shifts, and pattern complexity of anti-RR antibodies in a single-center retrospective study.

**Methods:**

A retrospective analysis was conducted on anti-RR-positive individuals identified from both the clinical cohort and the routine health checkup cohort. Clinical data, including disease categories, age, gender, concurrent ANA patterns, and antibody titers, were systematically evaluated.

**Results:**

Our findings demonstrate a heterogeneous clinical distribution of the anti-RR (AC-23) pattern across disease categories. Within the infectious disease group, hepatitis B virus (HBV) accounted for the largest proportion (36.59%), followed by hepatitis C virus (HCV) (24.39%), with a male predominance (M:F = 2.4:1, p<0.05). Among systemic autoimmune diseases, rheumatoid arthritis (RA) was the most frequently observed condition (25.64%), followed by connective tissue diseases (CTD) and autoimmune liver diseases (AIH/PBC). A distinct age- and sex-related distribution was observed, with higher frequencies in females aged 19–45 years, whereas a higher frequency was observed in males aged >65 years within the clinical cohort (p=0.045). Overall, anti-RR antibodies were predominantly detected at low titers (1:100–1:320). In addition, multiple immunofluorescence patterns, particularly speckled patterns, were more frequently observed in patients with autoimmune diseases, whereas isolated anti-RR patterns were more commonly identified in the routine health checkup cohort.

**Conclusion:**

Anti-RR represents a distinct ANA pattern associated with a broad clinical spectrum in the Chinese population, extending significantly beyond HCV to include HBV infection and various non-infectious conditions. The diverse clinical associations and the predominance of low-to-moderate titers suggest that anti-RR should be interpreted as a general indicator of systemic immune involvement rather than a specific disease marker. Clinical management should prioritize comprehensive evaluation based on the individual’s context rather than relying on anti-RR for specific disease screening.

## 1 Introduction

Antinuclear antibodies (ANAs) detected by indirect immunofluorescence (IIF) are fundamental tools in clinical immunology for the evaluation of autoimmune and immune-mediated diseases (1–3). ANA testing was performed using HEp-2 cell-based indirect immunofluorescence assay (HEp-2 IFA), which allows detection of antibodies targeting both nuclear and cytoplasmic antigens. Although commonly referred to as ANA testing in clinical practice, HEp-2 IFA detects a broad spectrum of anti-cell antibodies. Beyond classical nuclear staining patterns, a range of cytoplasmic patterns has gained increasing attention because of their potential clinical relevance (4). Among these, the rods and rings (RR) cytoplasmic pattern, referred to as anti-rods and rings (anti-RR) (5), has attracted particular interest due to its initial association with hepatitis C virus (HCV) infection and antiviral therapy (6). The rods and rings (RR) structures, also referred to as cytoophidia, are filamentous assemblies formed by metabolic enzymes such as inosine monophosphate dehydrogenase (IMPDH). Anti-RR antibodies were first described in patients treated with interferon-α and ribavirin for HCV infection (6). Subsequent studies suggested that the formation of RR structures is associated with altered activity and filament assembly of inosine monophosphate dehydrogenase (IMPDH) during antiviral responses (7, 8). However, the traditional view of anti-RR as a disease-specific serological marker has been increasingly challenged. Emerging evidence indicates that anti-RR antibodies may also be detected in individuals without HCV infection and across a broad range of clinical settings (9, 10). Case series and small cohort studies have reported anti-RR positivity in patients with other viral infections (9), autoimmune diseases (11), metabolic disorders (12), and even in individuals without apparent disease. These observations remain difficult to interpret because most studies are limited by small sample sizes, focus on single disease categories, or lack systematic comparisons across heterogeneous clinical populations (9, 13, 14).These findings indicate that the clinical implications of anti-RR antibodies are highly heterogeneous and extend far beyond the traditional HCV paradigm. To date, large-scale real-world studies characterizing the distribution, titer patterns, and clinical associations of anti-RR antibodies remain scarce. Whether the distribution and immunofluorescence features of anti-RR differ between clinically evaluated patients and individuals identified in routine health checkup settings remains insufficiently characterized. Clarifying these issues is essential for the appropriate interpretation of anti-RR positivity in clinical practice, as misclassification may lead to unnecessary diagnostic evaluation or incorrect attribution of disease relevance. Despite this paradigm shift, large-scale, real-world epidemiological studies evaluating the distribution of anti-RR antibodies across a comprehensive spectrum of diseases remain scarce. Most existing cohorts are limited in sample size or restricted to specific disease groups. Furthermore, while recent studies highlight the potential role of autoantibodies (such as anti-endothelial cell antibodies) as biomarkers of organ damage, the association between anti-RR antibody titers and clinical laboratory parameters—such as liver and renal function or hematological indices—has not been systematically characterized. Specifically, in regions like Central China, where HBV and tuberculosis (TB) are endemic (9), the seroprevalence and clinical significance of anti-RR antibodies in these infectious populations and concomitant immune-mediated diseases remain poorly defined.

To address these critical gaps, we conducted a large-scale, cross-sectional, retrospective study utilizing a cohort of 81,860 ANA-positive individuals. We systematically characterized the prevalence, titer distribution, and co-occurrence of nuclear and cytoplasmic staining patterns associated with anti-RR antibodies. The objective of this study was to compare the distribution of anti-rods and rings (anti-RR) antibodies between clinical patients and individuals undergoing routine health examinations, to assess whether anti-RR represents a disease-specific serological marker or a broader immunological phenotype. Moderate-to-high titers of anti-RR antibodies were observed in a subset of patients; however, due to the lack of longitudinal and clinical outcome data, their potential clinical relevance cannot be determined in the present study and requires further investigation.

## 2 Materials and methods

### 2.1 Study populations

A total of 81,860 ANA-positive serum samples were retrospectively collected and analyzed at the clinical immunology laboratory of Tongji Hospital, Tongji Medical College, Huazhong University of Science and Technology, China, between January 2017 and October 2024. All samples were tested for antinuclear antibodies using indirect immunofluorescence assay (IIF) as part of routine clinical practice. Based on the source of testing, subjects were divided into two cohorts. The clinical cohort included patients from outpatient and inpatient departments undergoing diagnostic evaluation. The routine health checkup cohort consisted of individuals undergoing preventive health screening at the hospital’s health management center. HEp-2 IFA testing was performed based on clinical indications (e.g., abnormal laboratory findings or physician request) rather than as universal screening of a strictly healthy individuals. Patients with documented diagnoses of autoimmune diseases, infectious diseases, or metabolic-related conditions were excluded from this cohort. For anti-RR-positive cases identified in the clinical cohort, clinical diagnoses were further classified into five major categories: autoimmune diseases, metabolic-related diseases, infectious diseases, neoplasms, and conditions without a definitive diagnosis. For patients with multiple diagnoses, classification was performed based on the primary clinical diagnosis as recorded in the medical records at the time of ANA testing. When multiple conditions were present, priority was given according to clinical relevance in the following order: autoimmune diseases, infectious diseases, metabolic-related diseases, neoplasms, and others. Cases without a clearly documented diagnosis or with insufficient clinical information were categorized as “unspecified.” Each patient was assigned to a single category to ensure mutual exclusivity for statistical analysis.

The study was performed in accordance with the relevant guidelines and regulations and approved by the ethical approval and consent of the Tongji Hospital, Tongji Medical College, Huazhong University of Science and Technology Ethics Committee (TJ-IRB202308129).

### 2.2 ANA IIF assay and definition of anti-RR

All samples were analyzed using the same commercial HEp-2 substrate kit (EUROIMMUN, Lübeck, Germany) throughout the study period, ensuring consistency in pattern recognition, including the detection of the AC-23 (rods and rings) pattern. No changes in assay platform or slide provider occurred during the study period. Serum samples were initially screened at a dilution of 1:100. Samples that tested positive at this dilution were further serially diluted to 1:320 and 1:1000 to assess semi-quantitative ANA titers.

Briefly, 27 μL of diluted serum was applied to an individual biochip containing ethanol-fixed HEp-2 cells with a reaction field measuring 5 × 5 mm. The slides were incubated in a humid chamber at room temperature (18-25 °C) for 30 minutes. After incubation, the slides were washed with phosphate-buffered saline containing Tween-20 (PBS-Tween) for at least 5 minutes and then air-dried. Next, 27 μL of fluorescein isothiocyanate (FITC)-conjugated anti-human IgG secondary antibody was added to each biochip. The slides were incubated for an additional 30 minutes under the same conditions. After a second wash and air-drying, the slides were mounted for evaluation by fluorescence microscopy. Fluorescence pattern recognition and titer evaluation were performed using the EUROPattern Microscope System (EUROIMMUN), which provides standardized image acquisition and computer-assisted interpretation. To ensure analytical accuracy and consistency between observers, all anti-RR patterns and titers were independently reviewed by two experienced laboratory technicians who were blinded to clinical information.

**Figure 1:**
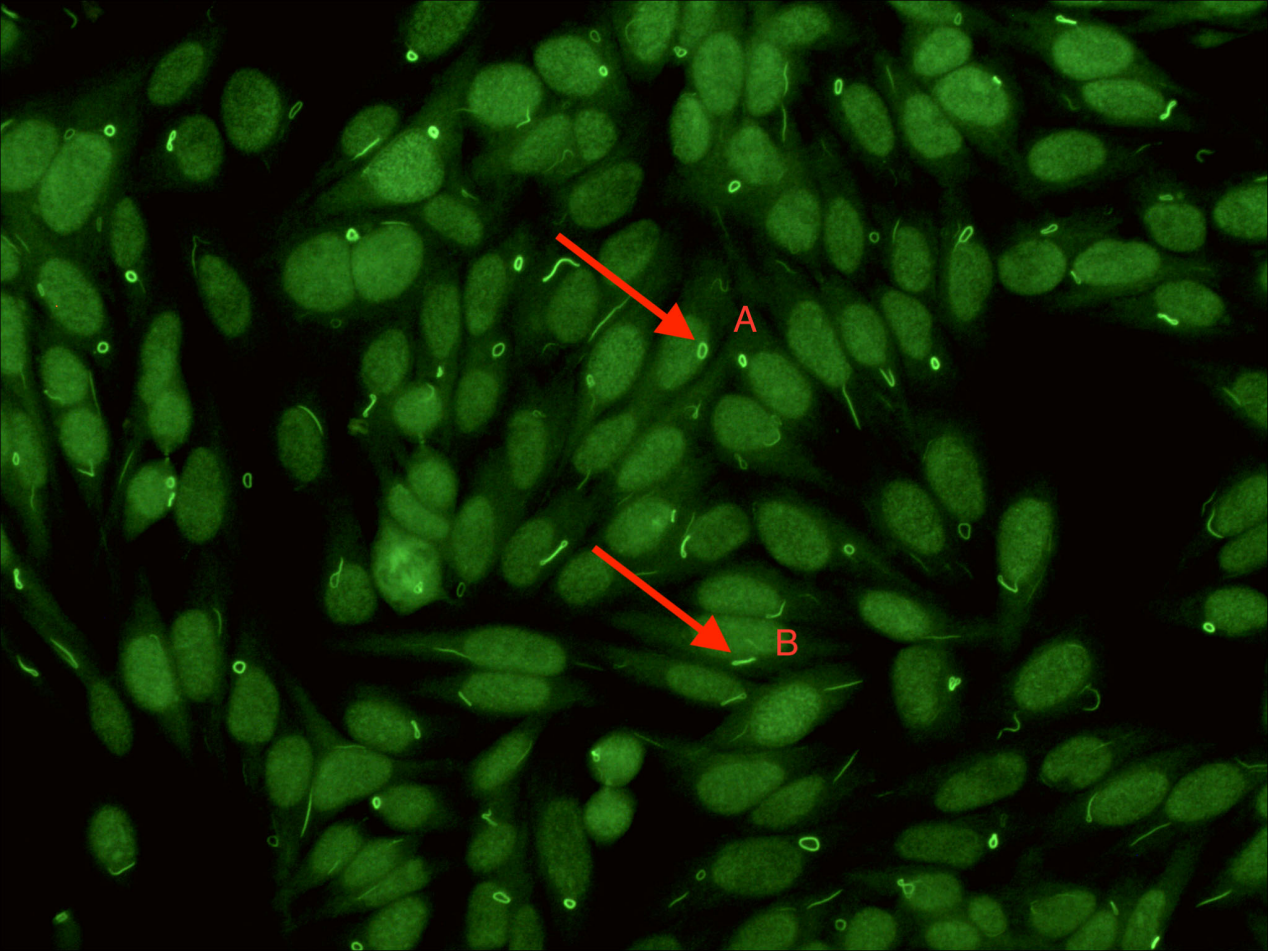
Indirect immunofluorescence pattern of anti-RR (AC-23). Arrows A and B indicate typical rod and ring structures, respectively. Visualization was performed on a EuroPattern system (EUROIMMUN) at 200× magnification using FITC fluorochromes. Image acquisition and analysis were conducted via EUROLabOffice software.

The rods and rings (RR) cytoplasmic pattern, corresponding to International Consensus on ANA Patterns (ICAP) AC-23, was identified by the presence of distinct rod- or ring-shaped cytoplasmic structures in HEp-2 cells (15). Representative immunofluorescence images of the anti-RR pattern are shown in **Figure 1**. Anti-RR positivity was defined as a clearly recognizable RR pattern at a serum dilution of 1:100 or higher, in accordance with the manufacturer’s recommendations and international consensus criteria. For descriptive and comparative analyses, samples that tested positive for anti-RR were stratified into three reactivity levels. These levels were defined as low (1:100), moderate (1:320), and high (1:1000 or greater), corresponding to serial three-fold dilutions within the validated dynamic range of the assay. These categories were used for semi-quantitative epidemiological analysis and should not be interpreted as diagnostic cut-off values.

### 2.3 Statistical analysis

Statistical analyses were performed using R software (version 4.3.2). Continuous variables were assessed for normality using the Shapiro-Wilk test. Normally distributed data are presented as mean ± standard deviation (SD) and were compared using the Student’s t-test, whereas non-normally distributed data are expressed as median with interquartile range (IQR) and were compared using the Mann-Whitney U test. Categorical variables are presented as counts and percentages and were compared using the chi-square test or Fisher’s exact test, as appropriate. Anti-RR titer distributions were analyzed using ordinal or categorical methods, and subgroup comparisons were conducted according to population source (clinical cohort vs routine health checkup cohort), disease category, and immunofluorescence pattern characteristics.

All statistical tests were two-sided, and a P value < 0.05 was considered statistically significant. Given the exploratory and descriptive nature of this retrospective study, no adjustments for multiple comparisons were applied unless otherwise specified.

## 3 Result

### 3.1 ANA-positive individuals: sex ratio and age distribution

In the disease group, 64,880 patients were ANA-positive, accounting for 79.26% of the total study population. The age of ANA-positive individuals ranged from 0 to 102 years. Among these patients, 20,632 were male, representing 17.19% of the total cohort. Their ages ranged from 0 to 98 years, with a mean age of 53.47 ± 18.20 years and a median age of 57 years. A total of 44,248 patients were female, accounting for 54.07% of the total cohort. Their ages ranged from 0 to 102 years, with a mean age of 49.03 ± 17.11 years and a median age of 52 years. The male-to-female ratio among ANA-positive patients in this group was 1:2.15. In the health check-up group, 16,980 individuals were ANA-positive, accounting for 20.74% of the total study population (16,980/81,860). The age range in this group was 12 to 100 years. Among them, 6,050 were male, representing 7.39% of the total cohort. Their ages ranged from 12 to 100 years, with a mean age of 46.2 ± 12.3 years and a median age of 51 years. A total of 10,930 were female, accounting for 13.35% of the total cohort. Their ages ranged from 12 to 93 years, with a mean age of 48.7 ± 13.1 years and a median age of 47 years. The male-to-female ratio in this group was 1:1.81. Comparison of the two cohorts demonstrated that ANA-positive individuals were predominantly female in both groups. In the disease group, male individuals were older on average than female individuals. In contrast, age differences between sexes were less pronounced in the health check-up group.

### 3.2 Prevalence, sex ratio, and age distribution of anti-RR-positive patients

In the disease cohort, 344 patients tested positive for anti-RR antibodies, yielding a prevalence of 0.53% (344/64,880) among ANA-positive individuals. Patient ages ranged from 0 to 90 years (mean: 55.08 ± 17.07). The group comprised 162 males (age range: 2–90 years; mean: 56.72 ± 16.92; median: 60.0) and 182 females (age range: 0–86 years; mean: 52.85 ± 16.84; median: 55.5), resulting in a male-to-female ratio of 1:1.12. A significant difference in age distribution was observed between the sexes in this group (Z = -3.018, P < 0.05). In the health check-up cohort, 105 individuals were anti-RR-positive, reflecting a prevalence of 0.62% (105/16,980). Ages ranged from 18 to 89 years (mean: 51.5 ± 12.5; median: 53.0). This group included 47 males (age range: 29–86 years; mean: 54.3 ± 11.1; median: 56.0) and 58 females (age range: 18–89 years; mean: 49.3 ± 12.9; median: 50.0), with a male-to-female ratio of 1:1.23.

Subgroup analysis by age (**Table 1**) revealed that in the 19–45 year bracket, the anti-RR positivity rate was significantly higher in females than in males (χ² = 6.12, P = 0.013). Conversely, in individuals over 65 years of age, the positivity rate was significantly higher in males (Fisher’s exact test, P = 0.045). In the 46–65 year bracket, positivity rates peaked for both sexes, with no significant difference between them (χ² = 2.89, P = 0.089). In summary, anti-RR positivity demonstrated distinct demographic trends, presenting predominantly in younger females (19–45 years) and older males (>65 years).

**Table 1:** Sex distribution of anti-RR–positive patients across age groups [n (%)].

Age (years)	Disease group (N=344)			Health check-up group (N=105)		
	Total	Male	Female	Total	Male	Female
≤18	14 (4.07%)	7 (2.03%)	7 (2.03%)	1 (0.95%)	–	1 (0.95%)
19–45	69 (20.06%)	23 (6.69%)	46 (13.37%)	30 (28.57%)	9 (8.57%)	21 (20.00%)
46–65	157 (45.64%)	72 (20.93%)	85 (24.71%)	66 (62.86%)	34 (32.38%)	32 (30.48%)
>65	104 (30.23%)	60 (17.44%)	44 (12.79%)	8 (7.62%)	4 (3.81%)	4 (3.81%)
Median Age		60.0 (2–90)	55.5 (0–86)		56.0 (29–86)	50.0 (18–89)
Mean Age		56.72 ± 16.92	52.85 ± 16.84		54.3 ± 11.1	49.3 ± 12.9

### 3.3 Distribution of anti-RR fluorescence titers and pattern characteristics

To further characterize the immunological profile of anti-RR antibodies, we analyzed the fluorescence titers and concurrent ANA patterns in both the health check-up and disease cohorts (**Table 2**). Overall, anti-RR antibodies were predominantly characterized by low titers across all subjects. In the health check-up cohort, the vast majority of cases clustered at 1:100 (75.24%) and 1:320 (21.90%), while high titers (≥ 1:1000) were exceedingly rare. A similar trend was observed in the disease group, where low titers (1:100 and 1:320) accounted for 70.93% and 22.09% of cases, respectively. Notably, however, the proportion of moderate-to-high titers (≥ 1:1000) was markedly higher in the disease group (6.97% combined) than in the health check-up group. While the isolated cytoplasmic RR pattern was frequently observed (51 cases in the health check-up group; 142 cases in the disease group), multiple patterns constituted a substantial portion of the anti-RR-positive landscape. Among these, the combination of anti-RR with a nuclear speckled pattern was the most prevalent in both cohorts (29 cases in the health check-up group; 108 cases in the disease group). Other common concurrent patterns included nuclear homogeneous, nucleolar, and cytoplasmic speckled patterns. Importantly, the disease group exhibited significantly greater diversity in staining profiles. Rare and complex combinations [including those involving the Golgi apparatus, nuclear dense fine speckled (DFS), spindle fibers, and Topoisomerase I (Topo I)] were identified exclusively in the diseased state. Additionally, a small subset of multiple patterns presented as triple-positive (e.g., concurrent with anti-intercellular bridge or anti-centromere antibodies), further highlighting the complex autoantibody signature associated with anti-RR in pathological conditions.

**Table 2:** Distribution of anti-RR fluorescence titers and pattern characteristics based on ICAP nomenclature [n (%)].

Pattern type	Disease group (N=344)				Health check-up group (N=105)			
	1∶100	1∶320	1∶1000	1∶3200	1∶100	1∶320	1∶1000	1∶3200
Isolated pattern								
Rods and rings (AC-23)	100 (29.07%)	31 (9.01%)	10 (2.91%)	1 (0.29%)	38 (36.19%)	12 (11.43%)	1 (0.95%)	0
Mixed patterns (Nuclear)								
Anti-RR + Nuclear speckled (AC-4, 5)	85 (24.71%)	18 (5.23%)	3 (0.87%)	2 (0.58%)	22 (20.95%)	6 (5.71%)	0	1 (0.95%)
Anti-RR + Nuclear homogeneous (AC-1)	11 (3.20%)	9 (2.62%)	4 (1.16%)	0	7 (6.67%)	5 (4.76%)	0	0
Anti-RR + Nucleolar (AC-8, 9, 10)	13 (3.78%)	3 (0.87%)	0	0	8 (7.62%)	0	0	0
Anti-RR + Nuclear discrete dots (AC-6, 7)	1 (0.29%)	2 (0.58%)	1 (0.29%)	0	0	0	0	0
Anti-RR + Nuclear envelope (AC-11, 12)	2 (0.58%)	3 (0.87%)	1 (0.29%)	0	0	0	0	0
Anti-RR + Dense fine speckled (AC-2)	1 (0.29%)	0	0	0	0	0	0	0
Anti-RR + Topo I (AC-29)	1 (0.29%)	0	0	0	0	0	0	0
Mixed patterns (Cytoplasmic)								
Anti-RR + Cytoplasmic speckled (AC-18, 19, 20)	22 (6.40%)	10 (2.91%)	1 (0.29%)	0	2 (1.90%)	0	0	0
Anti-RR + Cytoplasmic fibrillary (AC-15, 16, 17)	5 (1.45%)	0	0	0	2 (1.90%)	0	0	0
Anti-RR + Reticular/mitochondria-like (AC-21)	1 (0.29%)	0	1 (0.29%)	0	0	0	0	1 (0.95%)
Anti-RR + Polar/Golgi-like (AC-22)	1 (0.29%)	0	0	0	0	0	0	0
Mixed patterns (Mitotic)								
Anti-RR + Spindle fibers (AC-25)	1 (0.29%)	0	0	0	0	0	0	0
Total [n (%)]	244 (70.93%)	76 (22.09%)	21 (6.10%)	3 (0.87%)	79 (75.24%)	23 (21.90%)	1 (0.95%)	2 (1.90%)

### 3.4 Clinical classification and fluorescence titer profiles of anti-RR positive patients in the disease group

**Figure 2:**
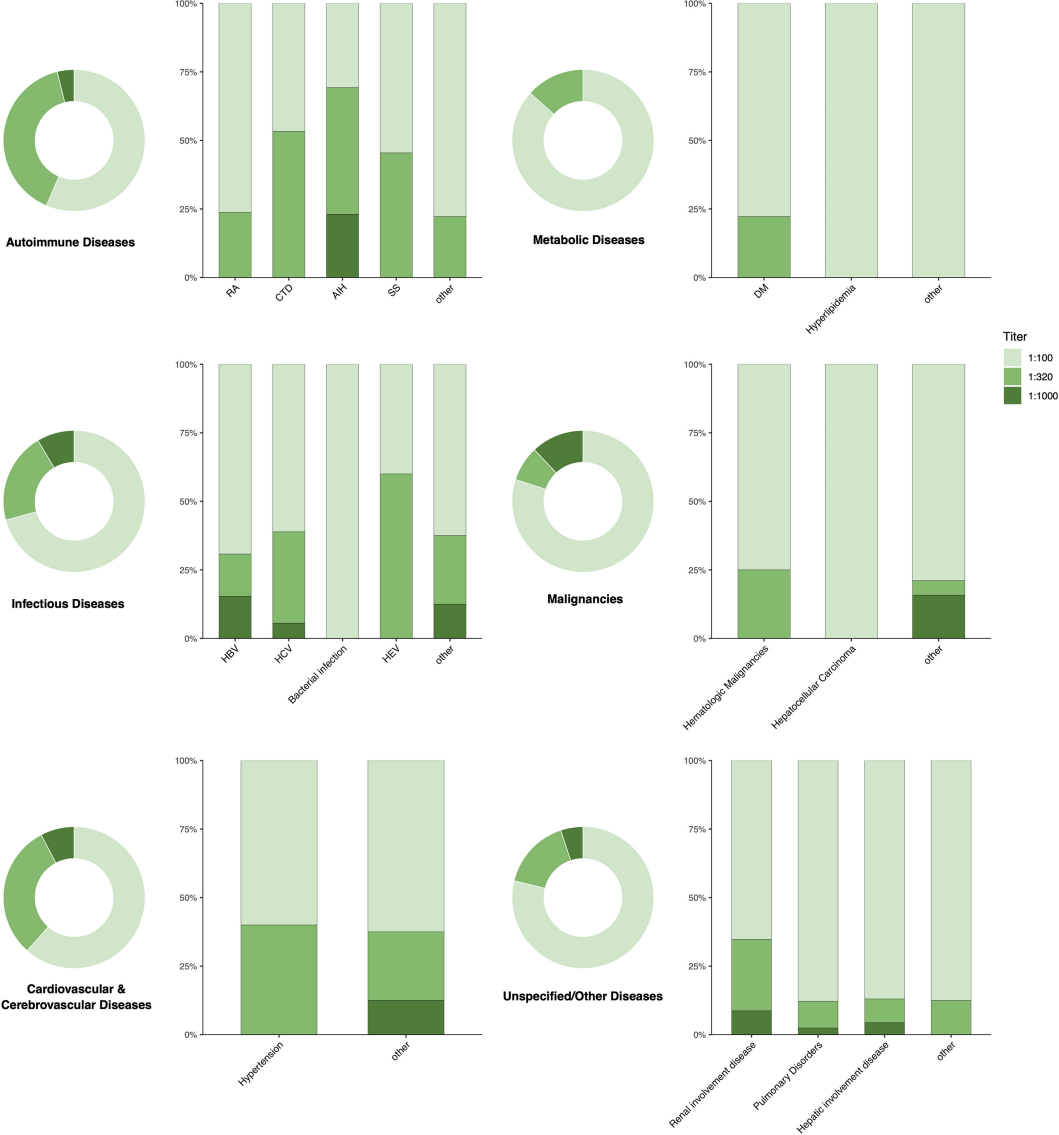
Distribution of anti-RR antibody titers and clinical sub-classifications across different disease categories. Clinical distribution and titer stratification of anti-RR antibodies. The cohort was categorized into six major diagnostic groups: Autoimmune Diseases, Metabolic Diseases, Infectious Diseases, Malignancies, Cardiovascular and Cerebrovascular Diseases, and Unspecified/Other Diseases. Left Panels (Donut Charts): Each donut chart represents the overall distribution of anti-RR antibody titers within the respective major category. The color gradient, ranging from light green to dark green, corresponds to the increasing titer levels: 1:100, 1:320, and 1:1000. Right Panels (Bar Plots): These stacked bar plots illustrate the proportional distribution of anti-RR titers within the top-ranked clinical sub-classifications for each group. The Y-axis represents the percentage (%) of patients, and the X-axis identifies specific diseases (e.g., RA, HBV, PBC, etc.). ANA, Antinuclear Antibody; AIH, Autoimmune Hepatitis; PBC, Primary Biliary Cholangitis; RA, Rheumatoid Arthritis; SS, Sjögren’s Syndrome; SLE, Systemic Lupus Erythematosus; CTD, Connective Tissue Disease; HAV/HBV/HCV/HEV, Hepatitis A/B/C/E Virus infection; EBV, Epstein-Barr Virus infection; TB, Mycobacterium tuberculosis (Tuberculosis) infection; RSV, Respiratory Syncytial Virus infection; CVD, Cardiovascular and Cerebrovascular Diseases; HBP, High Blood Pressure (Hypertension); AC-23, Anti-rods and rings immunofluorescence pattern (International Consensus on ANA Patterns).

Among the 344 anti-RR antibody-positive patients in the disease group, diagnostic disease classification was performed based on clinical records. The patients were categorized into 6 major groups: Autoimmune Diseases, Metabolic Diseases, Infectious Diseases, Malignancies, Cardiovascular and Cerebrovascular Diseases, and Unspecified/Other Diseases. The distribution of anti-RR antibody titers and their clinical sub-classifications across disease categories are presented in **Figure 2**. Among these categories, infectious diseases with 82 cases (23.84%), autoimmune diseases with 78 cases (22.67%), tumor-related diseases represented the smallest proportion with 25 cases (7.26%). Overall, among patients with confirmed diagnoses, anti-RR antibodies were most frequently observed in infectious diseases, autoimmune diseases, and metabolic-related diseases, while tumor-related diseases showed the lowest frequency of anti-RR positivity. Analysis of fluorescence titers across the different disease categories revealed that the majority of anti-RR antibodies were detected at low to moderate titers, predominantly ranging from 1:100 to 1:320. High titers (≥1:1000) were relatively uncommon. This distribution is consistent with previous studies reporting that anti-RR antibodies are typically detected at low or moderate fluorescence titers in routine ANA testing. Because the present study was retrospective and did not include longitudinal follow-up data, it was not possible to determine whether anti-RR antibody titers were associated with disease severity or disease progression. Nevertheless, anti-RR antibodies were frequently detected in patients with autoimmune diseases and infectious diseases, suggesting that the presence of anti-RR antibodies may be associated with immune activation or metabolic alterations occurring during disease processes.

Based on these findings, further subgroup analyses were performed for patients with confirmed diagnoses. Autoimmune diseases were further categorized into organ-specific and systemic autoimmune diseases, while infectious diseases were classified into viral infections, bacterial infections, and infections of unknown etiology in order to better characterize the distribution of anti-RR antibodies among different disease entities.

#### 3.4.1 Analysis of autoimmune diseases

Among the anti-RR antibody-positive patients in the disease group, 78 patients were diagnosed with autoimmune diseases, accounting for 22.67% of the disease cohort, with a male-to-female ratio of 1:1.29. These cases were further classified into organ-specific autoimmune diseases and systemic autoimmune diseases. Because this study was retrospective, only a portion of the cases had complete ANA profile testing results available. Among organ-specific autoimmune diseases, liver-related diseases were the most common. Autoimmune hepatitis was observed in 13 cases (16.67%), followed by primary biliary cholangitis in 4 cases (5.13%). In addition, kidney-related diseases included immune complex-mediated nephrotic syndrome in 3 cases (3.85%) and chronic kidney disease in 2 cases (2.56%). Pulmonary involvement was mainly represented by interstitial lung disease, which was identified in 7 cases (8.97%). Among systemic autoimmune diseases, rheumatoid arthritis showed the highest frequency with 20 cases (25.64%), followed by connective tissue diseases with 15 cases (19.23%). Other systemic autoimmune diseases included Sjögren’s syndrome (5 cases, 6.41%), systemic lupus erythematosus (5 cases, 6.41%), vasculitis (2 cases, 2.56%), and dermatomyositis (1 case, 1.28%).

These findings indicate that anti-RR antibodies in organ-specific autoimmune diseases were predominantly associated with liver diseases, whereas in systemic autoimmune diseases they were most frequently observed in patients with rheumatoid arthritis. In addition, among the patients who underwent ANA profile testing, anti-SS-A antibodies and anti-Ro-52 antibodies showed relatively high positivity rates.

#### 3.4.2 Analysis of infectious diseases

Among the anti-RR antibody-positive patients in the disease group, 82 cases were diagnosed with infectious diseases, accounting for 23.84% of the cohort. The male-to-female ratio was approximately 2.4:1, indicating a marked predominance of male patients. Further classification according to infection type revealed that viral infections accounted for the majority of cases with 62 patients (75.61%), followed by bacterial infections with 11 cases (13.41%) and infections of unknown etiology with 9 cases (10.98%). Among the viral infections, several hepatitis viruses were identified, including hepatitis A virus (HAV), hepatitis B virus (HBV), hepatitis C virus (HCV), and hepatitis E virus (HEV). In addition, infections with Epstein–Barr virus (EBV) and human immunodeficiency virus (HIV) were also observed. Among these viral infections, HBV infection accounted for the largest proportion of cases, followed by HCV infection. Previous studies have reported that anti-RR antibodies are closely associated with HCV infection, particularly in patients receiving pegylated interferon-α and ribavirin therapy. However, the findings of the present study indicate that anti-RR antibodies can also occur in other viral infections, with HBV infection representing the most common viral disease in our dataset.

In bacterial infections, anti-RR antibodies were most frequently observed in patients with Mycobacterium tuberculosis infection (7 cases, 8.54%), followed by infections caused by other pathogens such as Streptococcus, Legionella, and fungal organisms. Although the number of cases was limited, these findings suggest that anti-RR antibodies may also appear in non-viral infectious diseases.

## 4 Discussion

The anti-rods and rings (anti-RR) pattern, designated as AC-23 by the International Consensus on ANA Patterns (ICAP), has evolved from a laboratory phenomenon into a mandatory cytoplasmic pattern for ANA screening (16, 17). Historically, anti-RR antibodies were initially identified in patients with chronic hepatitis C virus (HCV) infection, particularly those undergoing combination therapy with pegylated interferon-α and ribavirin. The biochemical basis for this pattern involves the assembly of inosine-5’-monophosphate dehydrogenase 2 (IMPDH2) into macro-structures during metabolic stress. As IMPDH is the rate-limiting enzyme in guanine nucleotide biosynthesis, its inhibition—induced either pharmacologically by ribavirin or physiologically by rapid cellular proliferation (e.g., in activated T cells)—triggers RR formation (18). These observations suggest that the anti-RR pattern may extend beyond a purely drug-induced phenomenon and could reflect underlying cellular or metabolic stress across diverse clinical conditions, including those independent of HCV (18, 19). The accumulation of such structures within an inflammatory microenvironment may facilitate antigen presentation and contribute to a breakdown of immune tolerance, ultimately leading to the production of anti-RR antibodies (20). Previous studies have demonstrated that ribavirin is the primary agent inducing the formation of rods and rings structures through inhibition of IMPDH2, whereas interferon may act as a cofactor rather than the main inducer (21).

Large-scale retrospective studies have increasingly identified this pattern in a broad spectrum of conditions, ranging from autoimmune, renal, and pulmonary disorders to metabolic diseases and even asymptomatic individuals (10, 13, 14, 18). Such findings imply that anti-RR antibodies may reflect systemic alterations in cellular metabolism or immune activation rather than serving as a disease-specific marker. Nevertheless, our study provides robust evidence suggesting that the clinical landscape of anti-RR is significantly more expansive than previously recognized. In the present study, anti-RR antibodies were detected across multiple disease categories, supporting the concept that this antibody pattern is not limited to a single disease entity. A pivotal finding in our research is the significantly higher anti-RR positivity rate compared to previous domestic studies in Beijing, Inner Mongolia (22), and Dalian (13). We hypothesize that the COVID-19 pandemic serves as a major confounding factor. Our results resonate with the multi-center findings of İnal N et al (23), who observed a surge in anti-RR prevalence following the pandemic (p=0.00). SARS-CoV-2 infection is known to induce profound immune-metabolic disturbances. Recent studies have suggested a potential increase in anti-RR prevalence in the post-COVID-19 period. However, our study did not perform a time-stratified analysis, and therefore no direct inference regarding temporal trends can be made.

Another noteworthy finding of this study is the distribution of fluorescence patterns associated with anti-RR antibodies. Our results indicate that mixed ANA patterns were more frequently observed in patients with disease conditions, whereas isolated anti-RR patterns were more commonly detected in individuals from the health examination group. Among multiple patterns, nuclear speckled patterns were the most frequently observed, followed by cytoplasmic granular and homogeneous patterns. Previous studies have also reported that the RR pattern often coexists with speckled ANA patterns when multiple patterns are present (22). The coexistence of multiple ANA patterns may reflect broader immune activation or the presence of additional autoantibodies associated with systemic autoimmune processes. Importantly, the distribution of anti-RR across disease categories in this study reflects the composition of the underlying cohort rather than disease-specific prevalence. Therefore, these proportions should not be interpreted as the true prevalence of anti-RR within individual diseases. Regarding fluorescence titers, the majority of anti-RR-positive samples in our study showed relatively low titers, mainly ranging from 1:100 to 1:320, while high titers (≥1:1000) were relatively rare. This distribution is consistent with previous studies reporting that most RR patterns appear at low or moderate titers in routine ANA testing (22). Low titers may suggest that anti-RR antibodies often represent secondary immune responses associated with metabolic or inflammatory changes rather than primary pathogenic autoantibodies. Our data revealed that mixed nuclear patterns (particularly speckled patterns) were highly prevalent in diseased states, whereas isolated anti-RR was more common in health checkup cohort. While previous studies by Gu et al. suggested high titers (≥1:1000) are specific to AIDs, our cohort predominantly exhibited low titers (1:100-1:320) (13). This discrepancy could be attributed to variations in HEp-2 substrate sensitivity or differences in the patient recruitment threshold.

Our study identified a distinct demographic dimorphism in anti-RR distribution. Young to middle-aged females (19–45 years) exhibited higher positivity rates (χ2 = 6.12,p=0.013), likely reflecting the general predisposition of this demographic to autoimmune background. In the geriatric population (>65 years), males showed a disproportionately higher prevalence (p=0.045). These differences may reflect complex interactions between immune regulation, hormonal influences, and disease susceptibility. It is well known that females generally exhibit stronger humoral immune responses and have a higher prevalence of autoimmune diseases, particularly during reproductive age. Conversely, older males may have higher exposure to infectious or metabolic diseases, which may partially explain the increased anti-RR detection in this population. Particularly in the infection group, a striking 2.4:1 male-to-female ratio was observed. It may reflect the higher baseline prevalence of chronic viral infections in the male population. Given the retrospective design, sex-related differences may partly reflect variations in disease prevalence and healthcare-seeking patterns between males and females within the study population.

In our cohort, hepatitis B virus (HBV) accounted for a larger proportion of anti-RR-positive cases (36.59%) than hepatitis C virus (HCV) (24.39%). This difference may reflect variations in the underlying disease distribution rather than a disease-specific association. In regions such as China, where HBV is endemic, the interpretation of anti-RR findings should be considered within the local epidemiological context. An additional factor that may contribute to the observed differences from earlier studies is the evolving therapeutic landscape of HCV infection. Previous reports have consistently linked anti-RR positivity to HCV patients receiving ribavirin-based therapy, as ribavirin is known to induce polymerization of IMPDH2 into rods and rings structures. However, with the widespread adoption of direct-acting antiviral agents, ribavirin is now used far less frequently in clinical practice. In recent years, antiviral therapy for HCV infection has undergone substantial changes. In China, direct-acting antiviral agents have been widely adopted since approximately 2017–2018, largely replacing interferon- and ribavirin-based regimens. As ribavirin is a key inducer of RR structure formation, this transition in treatment strategies may partly explain the diminished association between anti-RR antibodies and HCV infection in more recent cohorts. This shift may partly explain why HCV is no longer the predominant condition associated with anti-RR in our cohort. In addition to ribavirin, several other medications, including mycophenolic acid, azathioprine, methotrexate, and acyclovir, have been reported to induce the formation of rods and rings structures through effects on nucleotide biosynthesis pathways (19). These findings suggest that drug-related mechanisms may contribute to the occurrence of anti-RR antibodies in a variety of clinical settings. However, due to the retrospective design of our study and the lack of detailed medication data, the potential contribution of specific drugs could not be systematically assessed and warrants further investigation. Furthermore, the association with Mycobacterium tuberculosis and various other viruses (HIV, EBV, HEV) suggests that anti-RR formation is a common downstream event of chronic infection-induced immune activation (22). Although we lacked detailed medication history (e.g., azathioprine or tenofovir), the prevalence of anti-RR in these non-HCV groups suggests that various hepatotropic viruses and their associated therapies may collectively contribute to the formation of the AC-23 pattern. Within the autoimmune disease (AID) spectrum, Rheumatoid Arthritis (RA) was the most prevalent condition (25.64%), mirroring findings in Turkish cohorts (23). This was followed closely by organ-specific autoimmune liver diseases (AIH) (22) and Primary biliary cholangitis (PBC) (12). Given that the liver is the primary metabolic hub, AIH-induced lymphocyte infiltration likely upregulates IMPDH activity, facilitating anti-RR expression.

Despite these novel insights, several limitations in our study must be acknowledged. First, as a retrospective analysis, there is an inherent selection bias, and establishing absolute causality between anti-RR production and specific viral or autoimmune triggers is challenging. Second, and most importantly, we lacked detailed clinical pharmacological data(e.g., specific dosages or duration of treatments like ribavirin, azathioprine, or antiviral agents for HBV). Consequently, we could not definitively isolate drug-induced RR formation from disease-driven metabolic stress. Lastly, the absence of longitudinal follow-up prevents us from evaluating the dynamic fluctuation of anti-RR titers in response to disease remission or progression. Given the retrospective and cross-sectional design of this study, no conclusions can be drawn regarding causality, disease progression, or prognostic significance. Future prospective and longitudinal studies are required to clarify the biological mechanisms and potential clinical implications of anti-RR antibodies.

## 5 Conclusion

In conclusion, anti-RR antibodies represent a rare but distinct cytoplasmic immunofluorescence pattern detectable across a broad range of clinical conditions beyond hepatitis C. In this ANA-positive cohort, anti-RR positivity showed heterogeneous associations with demographic characteristics, disease categories, and immunofluorescence patterns, with a predominance of low to moderate titers. Anti-RR antibodies were identified in both clinically evaluated patients and individuals undergoing routine health examinations, underscoring the need for cautious and context-dependent interpretation in clinical practice.

## Data Availability

The original contributions presented in the study are included in the article/Supplementary Material. Further inquiries can be directed to the corresponding author.
